# Comparison of analytical methods for the fatty acid profile in ewes’ milk

**DOI:** 10.1371/journal.pone.0263071

**Published:** 2022-02-16

**Authors:** Évelyn Silva de Melo Soares, Camila Celeste Brandão Ferreira Ítavo, Luís Carlos Vinhas Ítavo, Geraldo Tadeu dos Santos, Carlos Eduardo Domingues Nazário, Israel Shekinah Souza Soares, Leandro Fontoura Cavalheiro

**Affiliations:** 1 Animal Science Post-Graduation Program, Faculty of Veterinary Medicine and Animal Science, Campo Grande, Mato Grosso do Sul, Brazil; 2 Institute of Chemistry, Campo Grande, Mato Grosso do Sul, Brazil; University of Illinois, UNITED STATES

## Abstract

Studies comparing methodologies for fatty acids are very important, since they can influence the quality and final quantification of the lipid fraction. Objective—to compare different extraction methods for total lipids and to evaluate the effect of these methodologies on the quantitative composition of fatty acids in milk of lactating ewes raised in tropical pastures. The methodologies used were simple direct transesterification, using the HPLC grade organic solvent n-hexane, Bligh; Dyer (cold extraction, extracting mixture proportions 5, 10, and 15 mL in a ratio of 2:1:1 (v/v/v) of chloroform-methanol-water). The fatty acid methyl esters were separated by gas chromatography coupled with a flame ionization detector (GC-FID). The results show that there was no significant difference (p <0.05) in the total lipid content between the extraction methods. However, the Bligh’s method; Dyer obtained the best yield of lipids to be extracted from ewe’s milk, since the volume with 15 mL of the extraction solution was able to identify 87% of the chromatographic peaks. It was also observed that ewe’s milk has a higher percentage of palmitic, stearic and oleic fatty acids, with percentages of 20.1%, 15.5% and 33.1%, respectively. Therefore, an extraction mixture with a volume of 15 mL used in this study may be an alternative to perform the extraction of milk fat from high lactating ewes in tropical pastures as a routine method, as it expresses the best lipid content of this food.

## Introduction

Ewe’s milk has a chemical composition comprising proteins of high biological value and essential fatty acids, besides their mineral and vitamin content, which qualifies as a food of high nutritional value, also represents great importance in the diet for its characteristics of hypo allergenicity and digestibility because of the decreased fat cells [1].

However, ruminant milk fat contains high levels of saturated fatty acids (SFA), approximately 2/3 of total milk fatty acids [2], which are associated with several diseases in humans, in particular heart diseases. On the other hand, the presence of monounsaturated fatty acids (MUFA) and polyunsaturated acids (PUFA) and conjugated linoleic acid (CLA) in milk should be considered, as these are related to anticarcinogenic properties, reduction of atherosclerosis, among other beneficial effects on human health [1].

In this way, studies have been developed to evaluate the effects of the lipid fraction of milk and its derivatives on human health, since the determination of lipids is important for biochemical, physiological and nutritional studies [3]. The extraction of lipids is a critical stage in the analysis of total lipids, especially in the composition of fatty acids, since they suffer the interference from the seasons, the feeding provided to the animals, the lactation stage and food processing [4].

Therefore, methodological comparison studies for fatty acids are very important, because some samples require special care to get the lipid fraction, given that factors such as co-extraction of non-fatty component lipids and unwanted oxidation may influence the quality and final quantification of the lipid fraction [5].

In this sense, information on the nutritional quality of ewe’s milk is presented as a contribution to the search for alternatives for sheep farming and the strengthening of this production chain, since the literature on cow’s milk is constantly updated, whereas a few studies have been reported for ewes’ milk [2].

Therefore the aim of in this study compares different extraction methods for total lipids and evaluates the effect of these methodologies on the quantitative composition of fatty acids in milk of lactating ewes reared in tropical pastures, with the hypothesis of validating the technique that best expresses the total lipid content of milk from lactating ewes reared in tropical pastures, by providing a method that ensures the efficiency and reproducibility of the results, since this generates subsidies for standardization of method in several laboratories.

## Material and methods

The protocol described in this peer-reviewed article is published on protocols.io, https://dx.doi.org/10.17504/protocols.io.b3xsqpne, and is included for printing as S1 File with this article.

### Experimental and animal design

The milk samples used in this research were collected in the Sheep Farming Sector of the Federal University of Mato Grosso do Sul, at the School Farm of the Escola da Faculty of Veterinary Medicine and Animal Science. This experiment was carried out according to the ethical principles established by the National Experimental Control Council (CONCEA) and was approved by the Ethics Committee on Animal Use (CEUA) of the Federal University of Mato Grosso do Sul (UFMS—Protocol nº 1.119 / 2019).

The experimental trial occurred during the lactation phase (60 days), and the ewes were milked from the seventh to the 60th postpartum day, using the direct collection method [6]. During the morning period at 5:30 am, the lambs were separated from their mothers, who were destined for the first milking after administration of 1 mL of oxytocin (10 IU) intramuscular. Immediately after the injection, they were mechanically milked until no milk could be extracted from their udder. The sheep were returned to the pasture, while the lambs were kept in a separate corral. After 4 h, the procedure was repeated, the second milking was recorded and two representative milk samples (10 mL) were collected from each ewe for fatty acid analysis. Sixteen F1 Texel ewes (7 lambs with twin delivery and 9 single deliveries) were used, with a mean age of three, with a mean body score of 3 (scale 1 to 5) [7], with a mean postpartum weight of 64.36 ± 6.54 Kg. After milking, the ewes and lambs were returned to the pasture of Brachiaria brizantha cv. Marandu, where they had free access to water.

### Total lipid extraction

Total lipid extractions were performed according to the direct transesterification method (method 1), using the organic solvent n-hexane grade HPLC. Since method 1 was evaluated with extraction and derivatization in a single step, that is, directly (in situ), the other methods (method 1, 2 and 3) were performed with extraction and derivatization in different phases, using the [8] method, with chloroform, methanol and water (2: 1: 1), the proportions of 5 being evaluated, 10 and 15 mL of the extraction mixture, respectively. Method 2 was evaluated with 5 mL, whereas method 3 with 10 mL and method 4 with 15 mL of the extract mixture, respectively.

### Lipid fraction extraction methods

Total lipid extractions were performed in duplicate, and for a first direct attempt at extraction and derivatization (extraction method 1 –using HPLC grade n-hexane), the following steps were performed: in a 15 mL centrifuge tube with screw cap and Teflon septum, 1 mL of sample ewes’ milk was added to 2 mL of 0.5 M NaOH solution in methanol (chromatography grade), kept under heating in a water bath (60 C) for 5 min, and cooled to reach room temperature. Subsequently, 3 mL of the esterifying solution (15 mL H_2_SO_4_, 10g NH_4_Cl and 300 mL of methanol) was added, repeating the water bath process for 2 min and cooling at room temperature. Then, 2 mL of the saturated NaCl aqueous solution was added and agitated thoroughly 4 mL of hexane was added and vortexes again for 30 s. The solution was then centrifuged for 5 min at 2000 rpm and the supernatant removed, for it was where the formed fatty acid methyl esters (FAMEs) were found. After the procedure, the sample was used to analyze the FA profile by gas chromatography coupled to a flame ionization detector (GC-FID).

Through the simple method of isolation and purification described by [9], with adaptations made by [10], the extraction solvent was made using a cold mixture of chloroform–methanol–water extract in a ratio of 2:1:1 (v/v/v). This method was created by [8] and was performed: 1 mL of the milk sample was added to a 50 mL centrifuge tube with screw cap and Teflon septum, and 5, 10 or 15 mL of the extracting mixture was added, vortexed for 2 min and then placed on ultrasound for 20 min. Subsequently, 1 mL of chloroform, 0.5 mL of 2,0 M KCl solution was added and stirred for another 1 min in the vortex. After stirring, the tubes were centrifuged for 5 min at 2500 rpm. The lower layer containing the lipid extract was collected and evaporated under nitrogen flow and reserved for the derivatization step.

### Derivatization

After completion of the extraction process, the samples of methods 2, 3 and 4 (in proportions of 5, 10 and 15 mL of extracting mixture, respectively) were submitted to the derivatization process according to [11]. Into the glass tube containing the extracted lipid was added 2.0 mL of the 0.5 methanolic sodium hydroxide solution and it was kept under heating in a water bath (60ºC) for 5 min, and subsequently cooling at room temperature. Immediately 5.0 mL of esterifying reagent solution (15 mL H_2_SO_4_, 10g NH_4_Cl and 300 mL of methanol) was added, repeating the same heating method in the water bath at the same temperature, but left only 2 min, and cooled at room temperature again.

Then 2 mL of a saturated NaCl solution was added, and the content was agitated thoroughly 4 mL of hexane was used to extract the fatty acid methyl esters. The sample was then centrifuged for 5 min at 2000 rpm and an aliquot of the supernatant was removed and placed in a 2 mL bottle with a lid for further analysis by GC-FID. A summary of the experimental procedures for the extraction of lipids from ewe’s milk is shown in Table 1.

**Table 1:** Experimental procedures for the extraction of lipids from ewes’ milk.

Experiment	Ewes milk sample volume	Extraction solvent	Volume of solvent	Derivatization in situ
**Method 1**	1 mL	Hexane	4 mL	Yes
**Method 2**	1 mL	Chloroform-methanol-water 2:1:1 (v/v/v)	5 mL	No
**Method 3**	1 mL	Chloroform-methanol-water 2:1:1 (v/v/v)	10 mL	No
**Method 4**	1 mL	Chloroform-methanol-water 2:1:1 (v/v/v)	15 mL	No

### Analysis by GC-FID

The FAMEs were separated and determined by a gas chromatograph (Shimadzu®, model GC 2010) equipped with a fused silica capillary column (CP–BPX-70, 30 m × 0.25 mm, 0.25 μm) and flame ionization detector [12]. The carrier gas used was helium (purity of 99.999%). Injections were performed using an AOC 20i automatic injector equipped with a 10 µL syringe. The injected volume was 1 µL in 50:1 split mode, and the temperature of the injector and detector was set to 250°C. The column heating ramp was programmed to start at 80°C for 3 min, then increased at a rate of 10°C/min until reaching 140°C, thereafter increased to 5°C/min, until reaching 250°C, and remaining at this level for 5 min, totaling 40 min of analysis. Peak areas and retention times were determined using Shimadzu’s Lab solutions software. The identification of FAs was based on a comparison of retention times with the FAMEs standard.

The results were subjected to analysis of variance (ANOVA), and a comparison of treatment means was done by the Tukey test, adopting a significance level of 5%. Statistical analyses were performed using SAS Statistical Analysis Systems [13] version 9.0.

## Results

### Percentage area of FA profile

Table 2 shows the total lipid composition, the sum and the ratio between the groups of FAs in milk from lactating ewes raised on tropical pastures subjected to different lipid extraction methods. Bligh’s method; Dyer had the best yield among the analyzed methods, and method 4 (a method that uses derivatization and 15 mL of extraction mixture) was the one with the best yield when compared to method 1 (method in situ). In the lipid composition of milk, the average contents were 42.98% SFAs, 28.87% unsaturated fatty acids (UFAs), 26.21% MUFAs, 2.06% PUFAs and 0.68% (UFAs/SFAs).

**Table 2:** Percentage values of FA composition in ewes’ milk.

FAs	Method 1	Method 2	Method 3	Method 4	SEM	P
**C4:0 (butyric)**	1.07	1.10	1.65	1.49	0.02	0.2447
**C6:0 (caproic)**	0.68	1.20	1.25	1.18	0.03	0.5151
**C8:0 (caprylic)**	0.53 ^b^	1.05^a^	1.05 ^a^	1.05 ^a^	0.05	0.0091
**C10:0 (capric)**	1.60^c^	3.67^a^	3.15^b^	3.45^a^	0.02	<0.001
**C12:0 (lauric)**	0.82^c^	2.17^a^	1.55^b^	1.65^a^	0.02	<0.001
**C14:0 (myristic)**	2.79 ^c^	4.84 ^b^	4.65 ^b^	5.25 ^a^	0.03	<0.001
**C16:0 (palmitic)**	11.75^d^	16.03^c^	17.55 ^b^	20.05^a^	0.20	<0.001
**C16:1 (palmitoleic)**	0.40	0.74	0.85	1.06	0.14	0.1120
**C17:0 (heptadecanoic)**	0.67	0.63	0.75	0.86	0.11	0.5769
**C18:0 (stearic)**	9.47^d^	16.32^a^	13.35^c^	15.46^b^	0.08	<0.001
**C18:1n9c (oleic)**	20.67^c^	19.42^d^	27.05^b^	33.14^a^	0.06	<0.001
**C18:2n6c (linoleic)**	1.04^b^	1.93^a^	1.31^ab^	1.65^ab^	0.05	0.0014
**FAs identified**	53.47 ^d^	71.55 ^c^	76.75 ^b^	87.21 ^a^	0.70	<0.001
**Unidentified compounds**	46.53 ^a^	28.45 ^b^	23.25 ^c^	12.79 ^d^	0.37	<0.001
**Sums and ratio**						
**∑ SFAs**	29.38 ^d^	47.01 ^b^	45.37 ^c^	50.15 ^a^	0.38	<0.001
**∑ UFAs**	22.62 ^d^	24.55 ^c^	31.28 ^b^	37.02 ^a^	0.06	<0.001
**∑ MUFAs**	21.38 ^d^	20.53 ^c^	28.35 ^b^	34.59 ^a^	0.12	<0.001
**∑ PUFAs**	1.47 ^b^	2.64 ^a^	1.66 ^b^	2.46 ^a^	0.06	<0.001
**UFAs/SFAs**	0.77	0.52	0.69	0.74	0.04	0.3596

* Fatty acids (FAs) with percentage values less than 0.5% were not described in the table, but were accounted for in the total percentage of the quantification method. ∑SFAs = sum of saturated fatty acids; ∑UFAs = sum of unsaturated fatty acids; ∑MUFAs = sum of monounsaturated fatty acids; ∑ PUFAs = sum of polyunsaturated fatty acids; UFAs/SFAs = ratio between unsaturated and saturated fatty acids; SEM = mean standard error; Means followed by lowercase letters in the same column differ from each other by the Tukey test (P<0.05).

On the other hand, when comparing method 4 of this work with previous studies. It is possible to confirm that the ewes’ milk matrix has the FAs C16:0 (Palmitic acid), C18:0 (Stearic acid) and C18:1n9c (Oleic acid) as major components in their composition (Table 3).

**Table 3:** Percentage values of FA composition present in method 4 compared to previous studies.

FAs	Method 4	[12]	[14]	[15]	[16]
**C4:0 (butyric)**	1.49	1.18	1.43	8.00	1.60
**C6:0 (caproic)**	1.18	1.36	2.16	5.00	1.84
**C8:0 (caprylic)**	1.05	1.62	3.05	4.00	6.89
**C10:0 (capric)**	3.45	5.92	11.45	6.00	4.65
**C12:0 (lauric)**	1.65	3.99	7.46	5.00	2.58
**C14:0 (myristic)**	5.25	11.43	13.55	10.00	12.50
**C16:0 (palmitic)**	20.05	28.55	27.60	22.00	29.55
**C16:1 (palmitoleic)**	1.06	1.40	1.38	N.A.	1.20
**C17:0 (heptadecanoic)**	0.86	0.76	0.52	N.A.	0.70
**C18:0 (stearic)**	15.46	11.64	5.59	10.00	8.73
**C18:1n9c (oleic)**	33.14	19.64	16.19	22.00	20.19
**C18:2n6c (linoleic)**	1.65	2.22	3.74	4.00	2.66
**∑ SFAs**	50.15	67.78	74.42	N.A.	70.69
**∑ UFAs**	37.02	31.62	25.43	N.A.	29.32
**∑ MUFAs**	34.59	27.51	20.16	N.A.	24.63
**∑ PUFAs**	2.46	4.11	5.27	N.A.	4.69
**UFAs/ SFAs**	0.74	0.47	0.34	N.A.	0.41

* Fatty acids (FAs) with percentage values lower than 0.5% were not described in the table, but were accounted for in the total percentage of the quantification method. ∑SFAs = sum of saturated fatty acids; ∑UFAs = sum of unsaturated fatty acids; ∑MUFAs = sum of monounsaturated fatty acids; ∑ PUFAs = sum of polyunsaturated fatty acids; UFAs/SFAs = ratio between unsaturated and saturated fatty acids; NA = not identified.

## Discussion

### Determination of lipids or total fat

Although there are analytical methods for extracting lipids recommended for each class of food, these are under constant technical evaluation because of the need to consider the cost, extraction efficiency, toxicity, availability and quality of the final product [5]. Furthermore, due to its complexity, a complete analysis of all lipids present in the milk sample requires over one instrument and, therefore, the choice of this instrument depends on the study objectives [3].

Thus, based on the methodologies described by previous studies [12, 14–16], necessary modifications were made to the procedure for the extraction of lipids from sheep’s milk, as there were stages of the process where there were possibilities for improvement or the need to adapt to our laboratory routine.

In this sense, the experimental conditions for the extraction of lipids were based on different methods to determine the proportions of hexane and chloroform–methanol that would yield the greatest quantitative extraction of lipids.

In the in situ method, in which there is a single step of direct extraction and esterification of lipids, we observed the identification of 15 FAs, with oleic acid in the greatest proportion. However, this method identified an average of 53.47% of chromatographic peaks; this is because non-polar solvents (e.g., n-hexane) do not have the same efficiency in extracting lipids as polar solvents [17]. A second reason would be that the solubility of polar lipids is lower in hydrocarbon solvents, such as n-hexane, than in chloroform [17].

As for the different dilutions (5, 10 and 15 mL of the 2:1:1 (v/v/v) chloroform–methanol–water extraction solution) used in this test, it was observed that the percentage of identifying FAs increased with the volume of the extraction solvent. Thus, the method that used 15 mL of extraction solution identified on average 87.21% of the chromatographic peaks and presented the lowest percentage of unidentified compounds (12.79%).

This good yield observed for total lipids, provided by the method of Bligh and Dyer can be explained by the wide range of polarity presented by the mixture of solvents used. Thus, an advantage of the use of chloroform and methanol is that they are more polar than n-hexane and, thus, there is a more efficient extraction of polar and non-polar lipids [3].

### Gas chromatography coupled to flame ionization detector (GC-FID)

GC-FID is the most widely used separation and determination method, as it is easy to maintain and cheap (when compared to other detectors), and FAMEs can easily be identified and detected. The identification of FAMEs is made based on the elution order and retention time of these analysts [3].

However, GC-FID has a disadvantage, in that it is limited to volatile and thermally stable samples, so for non-volatile analysts, a previous derivatization step is necessary in order to transform them into volatile compounds [3]. Furthermore, this analysis can degrade lipids sensitive to high temperatures, which as a consequence may not be identified [3].

Based on this perspective, after the extraction process, samples from methods 2, 3 and 4 were subjected to derivatization, with the intention of the triglycerides being transformed into FAMEs. The programming was started at a temperature lower than the boiling point of the most volatile component present in the sample, after the heating rate of 10°C/min, and the second temperature level of 140°C assisted in the elution of fatty acid esters with an intermediate boiling point, that is, not as volatile as the previous ones. The final temperature used was 250°C to allow methyl fatty acid esters with a higher boiling point to be eluted [18].

In this way, through GC-FID it was possible to determine the chromatographic profile of the constituent fatty acids in sheep’s milk: about 68.65% of the total FAs found in the milk was palmitic acid 20.05%, 15.46% stearic acid and 33.14% oleic acid. The total of SFAs represented 50.15% of the total of FAs, while the UFA content was 37.02%. These results agree with other authors [19] who investigated the composition and distribution of lipids during lactation of Araucana native ewes reared in pasture systems. Through GC-FID, they found an average of 63.34% of SFAs, 31.91% of MUFAs, and 74.49% of the lipid composition comprised palmitic C16:0 (26.75%), stearic C18:0 (18.35%) and oleic C18:1n (29.39%) FAs, respectively, after 90 days of lactation.

When comparing method 4 (15 mL of extracting mixture) in this work with previous studies, it was possible to observe that the analysed milk samples had a greater level of UFAs. Similarly, the results obtained demonstrate that oleic acid (C18:1n9c) presented a higher percentage when compared to literature values.

The aim of this study was to compare different extraction methods for total lipids and evaluate the effect of these methodologies on the quantitative composition of FAs (through flame ionization detector response factors and internal standards) in milk from lactating sheep raised in tropical pastures and provide a standardized protocol and easy-to- for extracting total lipids from the milk of lactating ewes raised in tropical pastures.

## Conclusion

The methodology chosen for extracting the lipid fraction from the milk matrix of lactating ewes raised in tropical pastures may affect the yield of extracted lipids. In this study, the solvents used in the extractions influenced the extraction efficiency. Thus, based on the results of this work, when the lipid fractions were extracted, the use of 15 mL of extraction mixture was the one that presented the best results, which demonstrates the efficiency of the Bligh and Dyer methodology, since the extracts obtained with hexane presented a lower yield of total lipids in the analysed samples.

## Supporting information

S1 File (PDF)

S2 File (DOCX)
