# Supplementary material for: Comparison of analytical methods for the fatty acid profile in ewes’ milk
Source: PLoS One. 2022 Feb 16;17(2):e0263071. doi: 10.1371/journal.pone.0263071 (PMC8849528; doi:10.1371/journal.pone.0263071)
Supplement: S1 File — (PDF) [file pone.0263071.s001.pdf]

S1 file

Évelyn Silva de Melo Soares[1], Camila Celeste Brandão Ferreira Ítavo[1], Luís Carlos Vinhas Ítavo[1], Geraldo Tadeu dos Santos[1], Carlos Eduardo Domingues Nazário[2], Israel Shekinah Souza Soares[2], Leandro Fontoura Cavalheiro[2]

[1]Animal Science Post-Graduation Program, Faculty of Veterinary Medicine and Animal Science, Campo Grande, Mato Grosso do Sul, Brazil;

[2]Institute of Chemistry, Campo Grande, Mato Grosso do Sul, Brazil

dx.doi.org/10.17504/protocols.io.b3xsqpne

Luís Carlos Vinhas Ítavo

Studies comparing methodologies for fatty acids are very important, since they can influence the quality and final quantification of the lipid fraction. Objective - to compare different extraction methods for total lipids and to evaluate the effect of these methodologies on the quantitative composition of fatty acids in milk of lactating ewes raised in tropical pastures. The methodologies used were simple direct transesterification, using the HPLC grade organic solvent n-hexane, Bligh; Dyer (cold extraction, extracting mixture proportions 5, 10, and 15 mL in a ratio of 2:1:1 (v/v/v) of chloroform-methanol-water). The fatty acid methyl esters were separated by gas chromatography coupled with a flame ionization detector (GC-FID). The results show that there was no significant difference (p <0.05) in the total lipid content between the extraction methods. However, the Bligh's method; Dyer obtained the best yield of lipids to be extracted from ewe's milk, since the volume with 15 mL of the extraction solution was able to identify 87% of the chromatographic peaks.

DOI

dx.doi.org/10.17504/protocols.io.b3xsqpne

Évelyn Silva de Melo Soares, Camila Celeste Brandão Ferreira Ítavo, Luís Carlos Vinhas Ítavo, Geraldo Tadeu dos Santos, Carlos Eduardo Domingues Nazário, Israel Shekinah Souza Soares, Leandro Fontoura Cavalheiro . S1 file . **protocols.io** https://dx.doi.org/10.17504/protocols.io.b3xsqpne

protocol ,

Jan 17, 2022

Jan 17, 2022

57042

1 Step 1
Lipid fraction extraction methods
Total lipid extractions were performed in duplicate, and for a first direct attempt of extraction and derivatization (extraction method 1 – using HPLC-grade n-hexane), the following steps were performed: in a 15 mL centrifuge tube with screw cap and Teflon septum, 1 mL of sample ewes' milk was added to 2 mL of 0.5 M NaOH solution in methanol (chromatographic grade), kept under heating in a water bath (60°C) for 5 min, and cooled to reach room temperature. Subsequently, 3 mL of the esterifying solution (15 mL H2SO4, 10 g NH4Cl and 300 mL of metanol) was added, repeating the water bath process for 2 min and cooling at room temperature. Next, 2 mL of the saturated NaCl aqueous solution was added and agitated thoroughly 4 mL of hexane was added and vortexed again for 30 s. The solution was then centrifuged for 5 min at 2000 rpm and the supernatant removed, as this was where the formed fatty acid methyl esters (FAMEs) were found. After the procedure, the sample was used to analyze the FA profile by gas chromatography coupled to a flame ionization detector (GC-FID).
Through the simple method of isolation and purification described by [9], with adaptations made by [10], the extraction solvent was made using a cold mixture of chloroform–methanol–water extract in a ratio of 2:1:1 (v/v/v). This method was created by [8] and was performed: 1 mL of the milk sample was added to a 50 mL centrifuge tube with screw cap and Teflon septum, and 5, 10 or 15 mL of the extracting mixture was added, vortexed for 2 min and then placed on ultrasound for 20 min. Subsequently, 1 mL of chloroform, 0.5 mL of 2,0 M KCl solution was added and stirred for another 1 min in the vortex. After stirring, the tubes were centrifuged for 5 min at 2500 rpm. The lower layer containing the lipid extract was collected and evaporated under nitrogen flow and reserved for the derivatization step.

2 **Step 2**
Derivatization
To carry out the analysis of ewe's milk samples, the technique of GC-FID was used, with pre-treatment of sample by derivatization. The transesterification methodology was performed through the methylation of the lipid phase of the samples, according to the methodology [11].
After completion of the extraction process, the samples of methods 2, 3 and 4 (in proportions of 5, 10 and 15 mL of extracting mixture, respectively) were submitted to the derivatization process according to [11]. Into the glass tube containing the extracted lipid was added 2.0 mL of the 0.5 methanolic sodium hydroxide solution and it was kept under heating in a water bath (60ºC) for 5 min, and subsequently cooling at room temperature. Immediately 5.0 mL of esterifying reagent solution (15 mL H_2SO_4, 10g NH_4Cl and 300 mL of methanol) was added, repeating the same heating method in the water bath at the same temperature, but left only 2 min, and cooled at room temperature again. Then, 2 mL of saturated NaCl solution was added and the content was agitated thoroughly. 4 mL of hexane was used to extract the fatty acid methyl esters. The sample was then centrifuged for 5 min at 2000 rpm and an aliquot of the supernatant removed and placed in a 2-mL bottle with a lid for further analysis by GC-FID.

3 **Step 3**
Analysis by GC-FID
The methodology use dwas performed according to the AOCS MethodOfficialmethod described by the American Oil Chemists' Society(AOCS), in which the technique of GC-FID

[12].
The FAMEs were separated and determined by a gas chromatograph (Shimadzu®, model GC 2010) equipped with a fused silica capillary column (CP – BPX-70, 30 m × 0.25 mm, 0.25 µm) and flame ionization detector [12]. The carrier gas used was helium (purity of 99.999%. Injections were performed using an AOC 20i automatic injector equipped with a 10-µL syringe. The injected volume was 1 µL in 50:1 split mode, and the temperature of the injector and detector was set to 250 °C. The column heating ramp was programmed to start at 80 C for 3 min., then increased at a rate of 10 °C/min. until reaching 140 C, thereafter increased to 5 C/min, until reaching 250 C, and remaining at this level for 5 min, totaling 40 min of analysis. Peak areas and retention times were determined using Shimadzu's Lab solutions software. The identification of FAs was based on a comparison of retention times with the FAMEs standard.

4 **References**

8.Bligh EG, DYER WJ. A rapid method of total lipid extraction and purification. Canadian Journal of Biochemistry and Physiology, 1959, 27 (8): 911-917. doi: 10.1139 / o59-099.
9.Folch J, Lees M, Stanley GHS. A simple method for the isolation and purification of total lipids from animal tissues. The Journal of Biological Chemistry, 1957, 226 (1): 497-509. Available from: https://www.jbc.org/article/S0021-9258(18)64849-5/pdf.
10.Rule DC. Direct transesterification of total fatty acids of adipose tissue. and of freeze-dried muscle and liver with boron-trifluoride in methanol. Meat Science. 1997; 46 (1): 23-32.doi: 10.1016 / s0309-1740 (97) 00008-9.
11.Maia EV, Rodriguez-Amaya DBR. Evaluation of a simple and inexpensive method for the methylation of fatty acid with lipids of various fish species. Adolfo Lutz Institute Journal, 1993, 53 (1/2): 27-35. Available from: https://pesquisa.bvsalud.org/portal/resource/pt/lil-141017.
12.Aguilar C, Toro-Mujica1 P, Vargas-Bello-Pérez E, Vera1 R., Ugalde C, Rodríguez S. et al. A comparative study of the fatty acid profiles in commercial sheep cheeses.Grasas Aceites. 2014; 65 (4). 7. doi: 10.3989/gya.0460141
